# Impact of community-led cancer advocacy program on cancer health outreach and research navigation among immigrants of African descent

**DOI:** 10.3389/frhs.2026.1820297

**Published:** 2026-07-29

**Authors:** Opeyemi O. Bolajoko, Caleb O. Ramirez-Rivera, Olasanmi S. Bolajoko, Noreen A. Stephenson, Vinessa A. Gordon, Obianuju Aliche, Folakemi T. Odedina

**Affiliations:** 1 Cancer Health Equity Research Program, Mayo Clinic, Jacksonville, FL, United States; 2 Prokope Hills, Lagos, Nigeria; 3 Mayo Clinic Comprehensive Cancer Center, Jacksonville, FL, United States; 4 Department of Hematology/Oncology, Mayo Clinic, Jacksonville, FL, United States

**Keywords:** advocacy, cancer, community engagement, immigrants, research participation

## Abstract

**Introduction:**

Immigrants of African Descent (IAD) are a subset of the population in the United States with unique healthcare needs and experiences driving health disparities, including inadequate access to healthcare for cancer prevention and control. This study reports the impact of a Cancer advocacy program in IAD communities, using the training of trainers approach.

**Methods:**

IAD from different organizations were invited to undergo cancer advocacy training organized by Inclusive Cancer Care Research Equity and Mayo Clinic Comprehensive Cancer Center as Master trainers on colorectal, breast, and prostate cancers. Participation was through in-person training and Zoom sessions. Our team communicated with participants frequently via email to provide support and updates. The Master trainers led cancer awareness programs within their communities during the second phase of the program.

**Results:**

Participants included two breast cancer survivors, three prostate cancer survivors, six cancer caregivers, and 19 community advocates. There were 19 advocate-led cancer risk reduction and research participation events, reaching 2045 community members, and 875 were navigated to research. Community responses showed that 96% found the information given at the Master Trainer-led event was very useful, 46% promised to seek more cancer information, and 17% agreed to volunteer to become a cancer advocate. The insights from this study suggested that the train-the-trainers’ approach was efficient in ensuring community ownership of cancer advocacy.

**Conclusion:**

IAD can be trained to effectively lead advocacy programs in immigrant communities, using the train-the-trainer approach. Future studies should establish the feasibility of sustaining community outreach and research navigation in IAD communities to improve cancer prevention and cancer care outcomes in this population.

## 1 Introduction

Men of African Ascestry (MoAA) endure a disproportionate burden of cancer, impacting their morbidity and mortality (1). This includes immigrants in the United States. With a growing population, Sub-Saharan Africa (SSA) and the Caribbean are the largest subregional origins (2). Immigrants have unique healthcare needs and experiences, but are usually grouped with the general MoAA in the United States (3). Despite evident gaps, this group is the least studied immigrant group (2), experiencing limited health programs and research targeting their communities (3, 4). Insufficient data on this population creates barriers to making evidence-based, critical healthcare decisions and policies for immigrants of African descent (IAD). Other challenges in healthcare for IAD include ineffective integration into the community (5), an ineffective acculturation process, racial (4), rural-urban (6) cancer literacy (7), and other social determinants of migrant health, including stigma and marginalization, acculturation difficulties, and sometimes fear of deportation if undocumented (8). Inadequate access to healthcare to cover the cost of cancer care and control is the bane of most immigrants, since they are excluded from most government initiatives and programs based on citizenship. Some, especially when undocumented, often face significant health challenges, including higher prevalence of chronic diseases, lower access to quality healthcare, and poorer health outcomes (9).

Training cancer advocates from the immigrant communities is a promising implementation approach to addressing misinformation and stigma surrounding cancer, especially in medically underserved populations (10). Meaningful inclusion of diverse populations is important to ensure evidence-based cancer prevention programs, diagnosis, and treatment decisions are equitable and responsive to diverse communities (10). Cancer advocates advance the priorities of the community they serve and ensure that the needs of their communities are met through community-driven efforts. They raise cancer awareness and education in the language their community members understand. This empowers individuals to take control of their own health through adopting lifestyle changes that reduce cancer risk. Advocates work with other stakeholders to ensure the adoption and sustainability of cancer prevention, early detection, treatment, and survivorship initiatives. Earlier works have provided evidence of the roles of advocates in educating community members to gain knowledge about cancer risk factors, the importance of screening and preventive measures, and navigation to healthcare system resources (11, 12). Training cancer advocates in IAD communities may strengthen trust in the healthcare system, improve dissemination of evidence-based cancer information, and enhance the contextual implementation and adoption of interventions.

The IAD population has inadequate representation in bio-behavioral and clinical research, which limits the generalizability of findings (10). Dissemination of evidence-based cancer information, health promotion, and advocacy are important in advancing cancer prevention, increasing screening uptake and early detection, as well as encouraging research and clinical trial participation. Cancer advocates play a vital role in bridging the gap between patients and providers, as well as investigators and research participants (10, 13). More importantly, the problem of research recruitment and retention continues to exacerbate health outcomes in these populations (10). The factors determining research recruitment and retention have been well researched and are known to be influenced by both patient-related barriers/motivators and physician-related barriers/motivators. When cancer advocates engage in research, they ensure that the voices of these communities are heard and research is tailored to their specific health needs. Research advocacy then strengthens these efforts by increasing access to research and fostering trust between investigators and community members (13). By combining education with community-driven initiatives, we can enhance early diagnosis, improve health outcomes, and reduce the overall burden of cancer. Thus, establishing a robust network of research advocates in communities is crucial for health programs’ implementation, building the premise for trust, and ensuring the sustainability of engagement in scientific research.

In response to address these needs, the Inclusive Cancer Care Research Equity (iCCaRE) Consortium and the Office of Community Outreach and Engagement at Mayo Clinic Comprehensive Cancer Center collaborated with the African Immigrant Coalition for Health to advance an all-inclusive initiative that would close the disparity gap among the IAD communities in the United States. The long-term goal of this engagement is to eliminate the disparities in cancer care and control, reduce morbidity and mortality through continuous community engagement and cancer advocacy programs, research participation, and culturally appropriate interventions integrated into the existing structure and infrastructure of IAD communities. The study's primary aim was to assess the community impact of trained research and cancer advocates who were empowered to engage their communities, implement cancer risk reduction programs, and provide support to investigators who seek to include IAD in their studies. The central hypothesis for this program was that community-driven cancer advocacy and literacy and navigation services by community stakeholders would significantly improve cancer awareness and research participation among IAD.

## 2 Methods

### 2.1 Study setting

As a first step in achieving our long-term goal of making cancer a priority in the African and Caribbean immigrant community, leaders were invited to partner with investigators at Mayo Clinic Florida to brainstorm on the current state of health and wellness in IAD communities and the need to prioritize cancer advocacy. The importance of participating in research was also reiterated. A four-hour listening session was organized, and leaders of these communities participated. The listening session started with a presentation on the Mayo Clinic's and iCCaRE's current cancer control initiatives in the community. The presentation highlighted how community organizations could partner to advance health initiatives, cancer care, and control in the community. Some of the initiatives and collaboration opportunities were shared, including the Citizen Scientist and Cancer Advocacy programs. It was further explained that the two initiatives are aimed at engaging members of the community with the desire to advance cancer advocacy and control, as well as cancer research and clinical trials in the communities. The community leaders expressed interest in partnering with the iCCaRE Consortium and the community outreach and engagement unit of the Mayo Clinic Comprehensive Cancer Center for a cancer advocacy training program targeted at IAD.

### 2.2 Study design and framework

We implemented the “***Cancer Advocacy Training for Immigrants of African Descent Communities***,***”*** based on a community-based participatory program using the training of trainers (ToT) implementation strategy. This approach is used in implementation science to empower targeted community members as local stakeholders to facilitate the adoption and sustainability of evidence-based interventions in real-world settings (14). As such, participants were empowered with the skill set needed to lead advocacy programs in their respective communities. The transfer of knowledge and skills is a central goal of ToT interventions. The community-led advocacy program leveraged the lived experiences and cultural insights of IAD leaders, cancer survivors, caregivers, and community influencers to drive implementation and adoption of the cancer awareness campaign process.

Utilizing a health belief model (15), the workshop goal was to train cancer advocates to: (1) mobilize the resources within the IAD communities for cancer prevention strategies and risk reduction, (2) partner with key stakeholders to advance cancer awareness programs, and provide support for research activities; (3) raise funds to support advocacy activities; and (4) develop and successfully organize community-centered programs.

### 2.3 Recruitment

The recruitment process included a promotion flyer with information about the details of the program, the start date, the duration, how to apply distributed through [1] the use of Mayo Clinic and iCCaRE Consortium websites for program information and registration; [2] direct emails to leaders of IAD communities for the nomination of eligible members in their communities; [3] targeted emails to African and Caribbean immigrant communities through community organizations databases; and [4] direct mailings of training information to cancer centers and academic institutions. Interested individuals contacted the team members listed for more information or assistance with applying for the program. Participants for this program were selected through a rigorous screening process based on: (i) interest and past involvement in advocacy, (ii) personal commitment to cancer advocacy, (iii) tangible goals, and a feasible plan to engage a minimum of 50 community members within 3–4 months; and (iv) evidence of community organization's commitment to meet advocacy training goals, through support letters.

### 2.4 Training expectations

The learning objectives for the training sessions were to: (a) Assess and track the cancer health needs of IAD; (b) Initiate and develop partnerships with other stakeholders to address the needs of their communities; (c) Organize and implement programs to: [i] raise public awareness about cancer; [ii] provide support to those living with cancer; [ii] help advance cancer research and training; and (iii) Utilize evaluation tools to determine, document, and improve the program outcomes of programs implemented in their communities.

### 2.5 Training phases

The training was carried out in two phases.

### 2.6 Phase 1 master trainer workshop

#### 2.6.1 Training resources and materials

Phase 1 was focused on training Master trainers in one of the following advocacy areas: Political, Research, Fundraising, Support, and Community Outreach. The cancer types covered were colorectal cancer, breast cancer, and prostate cancer. The training resources and materials for the program included a Cancer Advocacy manual, which was first published in 2019 and updated by the Mayo Clinic Comprehensive Cancer Center and the iCCaRE consortium in 2024. The updated manual covers more cancer topics and best practices for advocacy, including cancer advocacy plan, political, support, research, fundraising, community outreach, and clinical trial advocacy. In addition, access to the web-based self-paced Cancer Awareness and research engagement modules was provided to the Master Trainers for more training on research advocacy and participation in clinical trials. Master trainers were also provided with prostate health information flyers for distribution during advocacy. All materials were provided in the English language. Participants were IAD from different community-based and faith-based organizations across different cities in the United States, including Nigeria, Ghana, Cameroon, Liberia, South Africa, Haiti, and Jamaica.

#### 2.6.2 Advocacy training

Each Master Trainer was funded to attend two-day advocacy training sessions, and follow-up was through in-person meetings and Zoom sessions based on the needs and availabilities of each advocate. They were asked to choose any of the three specialized cancer advocacy sessions and were trained and mentored by specialist advocates and mentors, including established community cancer advocates and scientists. They were provided a trainer manual to assist them in cancer advocacy and community research engagements. The advocacy focus for the Master trainers includes (i) awareness and education about either colorectal, breast, or prostate cancers, (ii) the need to go for routine screening in line with guidelines, and (iii) the need for research participation and clinical trials. They were encouraged to engage at least 50 community members and were also required to submit progress and outcomes of their cancer advocacy efforts. Participants passed the program assessment before being certified as Master trainers.

#### 2.6.3 Mentored sessions

After the specialist training, each Master Trainer was paired with one established advocate and one faculty member who served as mentors to guide the second phase of the program. Through bi-monthly meetings, mentors supported the planning, implementation, and evaluation of the cancer advocacy programs by the Master trainers. Additionally, our team communicated with participants frequently via email, online meetings, and in-person meetings when necessary, to provide support and updates. The Master trainers worked with assigned advocates and faculty mentors to develop and design a tailored advocacy program for their communities.

### 2.7 Phase 2 master trainers-led outreaches

Phase 2 focused on helping participants identify an advocacy project; create their teams; research information; design, implement, and evaluate advocacy projects; and report findings, experiences, and lessons learned from conducting the advocacy project. Master Trainers-led cancer advocacy programs were carried out as part of their experiential and hands-on training. We worked with the Master Trainers to engage at least 50 community members in either colorectal, breast, or prostate cancer advocacy within 6–8 months. They were compensated for their advocacy efforts and cancer awareness campaigns. In addition, during this program, Master trainers were offered opportunities to co-lead engagement in research by navigating community members interested in research to available studies if eligible; those who volunteered to serve as community leads for research were compensated based on the budget of those studies. Available studies and programs include the Mayo Clinic Community Research Registry, the cooked meat study, and the digital literacy program, among others.

Moreover, their community engagement efforts include cancer awareness events, engagement of clients at community pharmacies, African stores, restaurants, etc. Community members who attended those events and were interested in participating in research were also navigated to available research if eligible.

### 2.8 Program evaluation

Evaluation was carried out to determine the success of the Afri-CAT program based on the anticipated deliverables and expected outcomes as follows: (1) The number of Master trainers trained to lead cancer advocacy and research, from the IAD communities; (2) the number of individuals reached and engaged for cancer advocacy, measured through the number of attendees in the programs; (3) the number of individuals who participated in research through the number of accruals to research, which were facilitated by the direct efforts of the Master trainers. An evaluation survey was used to obtain feedback from community members who participated in the Master trainer-led advocacy program. The question asked included the usefulness of the cancer awareness information received, the probability they will seek more health information on cancer, how they intend to use the information they received in the near term, and if they intend to participate in research.

### 2.9 Data analysis

Descriptive statistics were used to summarize demographic information of Master trainers and information obtained from the program evaluation survey. Frequency and percentages were used to summarize the number of events, participants reached, and community members who participated in research through the direct efforts of the Master trainers. Comments were obtained from the evaluation sheets of those who provided feedback after the programs led by the Master trainers. However, we could not comprehensively analyze the feedback because not all event attendees provided written feedback.

### 2.10 Ethical approval and informed consent statements

The advocacy program was considered exempt by the Institutional Review Board of Mayo Clinic (Approval number: 22-005333). All participants volunteered to be part of the cancer advocacy training and to lead cancer prevention campaign events in their communities.

## 3 Results

A total of 30 participants underwent the cancer advocacy training program, with the majority male at 52%. Regarding educational attainment, nearly half of the participants had an Associate degree (46.7%). All participants joined the program through nomination and/or recommendation. Participants were drawn from immigrant churches and community organizations across different ethnicities, including Nigeria, Ghana, Cameroon, Liberia, South Africa, Haiti, Jamaica, and across different cities in the United States. Most of the advocates reside in Florida (70%), with 7.4% from Texas, 11% from Minnesota, and 7.4% from New Jersey. Participants included two breast cancer survivors, three prostate cancer survivors, six cancer caregivers, and 19 community advocates. The majority (74%) self-identified as African immigrants, while 26% were Caribbean immigrants. Advocates were categorized into three working groups, including prostate, breast, and colorectal cancer advocacy groups (Table 1).

**Table 1:** Demographics of participants of the master trainers.

Countries	No.	%
Gender		
Females	13	43.3
Males	17	56.7
Age (years)		
30–39	5	16.7
40–49	3	10.0
50–59	10	33.3
≥60	12	40.0
Race		
African immigrants	22	73.3
Caribbean immigrants	8	26.7
Education Level		
High school	5	16.7
Associate degree	14	46.7
Bachelor's degree	3	10
Vocational training	1	3.3
Graduate degree	7	23.3
Advocates Categories		
Breast cancer survivor	2	6.7
Prostate cancer survivor	3	10
Cancer caregivers	6	20
Community influencers	19	63.3
Residence		
Florida	21	70
Texas	4	13.3
Minnesota	3	10
New Jersey	2	6.7
Countries of origin		
Jamaica	2	6.7
Nigeria	13	43.3
Cameroon	5	16.7
Liberia	1	3.3
Haiti	6	20
Kenya	1	3.3
Somalia	2	6.7

Table 2 shows advocate-led community engagements for cancer risk reduction and research participation events, reaching about 2,045 community members, and 875 have already been navigated to the Mayo Clinic research. Master trainers’ community engagement and cancer programs included 15 conversations about cancer risk reduction practices in churches and ethnic groups, three virtual campaigns, and master trainers co-led recruitment activities in four States in the United States.

**Table 2:** Master trainer-led cancer campaign outcomes.

Research and programs	Number of events	Number of participants
Cancer and health literacy programs		
In-person cancer campaigns	9	1,270
Digital literacy	1	10
Prostate health dialogues	6	118
Virtual and online cancer campaigns	3	647
Total	19	2,045
Research studies participation		
Research registry	6	521
Cooked meat study	4	252
Caregiver Study	2	2
Perception about decentralized trials	2	100

**Figure 1:**
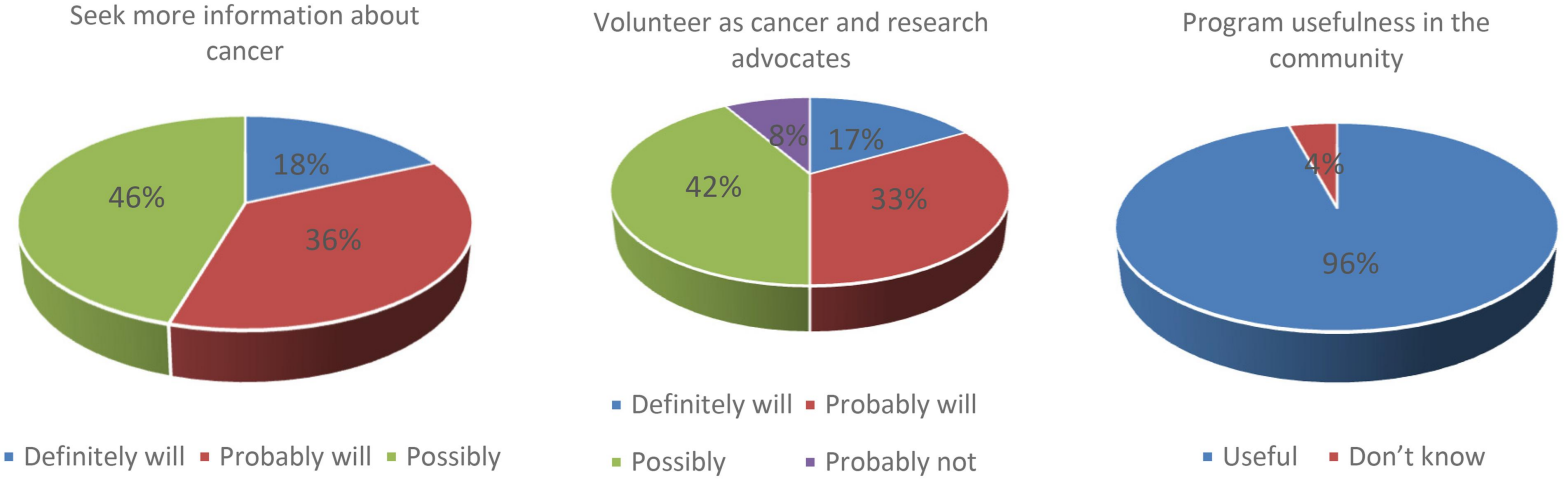
Participants’ feedback on master trainer-led cancer advocacies.

The responses from the community members were summarized in Figure 1. The majority (96%) of the community members who responded to the evaluation surveys reported that the information presented during the Master Trainer-led events was very useful. About half (46%) reported an intention to seek additional cancer information, and 17% reported that they will definitely volunteer for research, while 75% were cautiously positive about their intention to participate in research.

Table 3 shows some of the feedback from community members who attended some of the advocacy cancer awareness campaigns led by Masers Trainers. Some expressed their gratitude and promised to spread the word.

**Table 3:** Selected comments and feedback.

Comments and feedback
“The information is very good; I would like you back again.”
“The presentation was very informative- Definitely, I want to get involved and participate.”
“You have done a great job, really explicit. Thank you.”
“Highly informative, educative presentation, good job.”
“I have four brothers. I will tell them the information I learned and have them visit the website.”
“I will encourage family, friends, and neighbors to screen for CaP.”

## 4 Discussion

This cancer advocacy program, developed for IAD, was based on community input to demonstrate the feasibility of the ToT approach for building cancer advocacy capacity among IAD. A diverse group of community members, including cancer survivors, caregivers, and community advocates, was trained to lead cancer education and awareness initiatives in their communities. One of the indicators of successful implementation was that trainees were not merely recipients of information but became active implementers of the intervention within their communities through Master trainer-led community outreach events, reaching community members and connecting eligible individuals to research opportunities.

The ToT approach provided an equitable opportunity for community stakeholders to lead cancer advocacy programs, thus placing advocacy efforts in the hands of the stakeholders of the community of interest. An earlier study reported ToT approach to be a scalable and sustainable method for expanding advocacy capacity and increasing health education and promotion within communities (16). The success of the program lies in grassroots mobilization and inclusiveness, which gives members of the community equitable opportunities to have a voice and sustainable program outcomes. Selected individuals were equipped with specialized training on leading advocacy and research recruitment in their communities. These advocates facilitated, mobilized, and led group cancer awareness programs, cancer conversations, and one-on-one biomedical education and cancer risk reduction engagement with community members, creating a multiplier effect. Earlier studies have reported that engaging individuals from the target communities could help build trust in research institutions among minority groups (13, 17). The master trainers and advocates became the trusted intermediaries, facilitating culturally responsive communication, countering misinformation about cancer risk and research participation. This ToT approach ensured that advocacy knowledge is disseminated efficiently and continuously within the community, fostering long-term engagement and enhancing the overall reach of cancer education and prevention initiatives (16).

Three cancers, including prostate, breast, and colorectal cancers, were prioritized for advocacy in this program. Prostate cancer is the second most common cancer among men (18). Studies have shown that the disproportionate burden of CaP borne by MoAA might be due to inadequate screening and detection at late stages (19, 20). In addition, the mortality of prostate cancer is high among MoAA due to late presentation at advanced stages of the disease (21). Similarly, breast cancer was prioritized as the most common cancer among women, with more aggressive types among women of African ancestry (22). Unfortunately, these are diagnosed at late stages due to poor uptake of screening among these women, including immigrants (23). Colorectal cancer was also prioritized because the prevalence is currently rising in MoAA communities, and it is one of the most common cancers affecting males and females (24, 25).

Community-led outreach strategies demonstrated adoption of the advocacy model by trained participants. It enhanced the engagement of community members for cancer awareness programs, resulting in substantial reach. Our program featured several cancer advocacies, cancer literacy and prevention programs, as well as recruitment into research studies led by the advocates. This might be because the advocates have a good understanding of cultural nuances, local concerns, and potential barriers to participation (13). Given the limited representation of immigrants in cancer prevention initiatives and clinical research, the reach of this program through community-led activities suggests that the ToT model may be effective for expanding access to preventive health initiatives and research opportunities among IAD. Research advocates drawn from communities serve as trusted intermediaries between investigators and community members, facilitating communication (13). The program's model suggests the development of a sustainable advocacy workforce development in which community members transition from information recipients to co-lead cancer control efforts.

In addition, the Master Trainers were paired with faculty mentors with specialization in the cancers of interest for oversight of program development and research engagement. They also had survivor advocates in prostate, breast, and colorectal cancers who supported them with planning advocacy programs in their respective communities. Mentorship could ensure that advocates are focused on the objectives of the program, as well as lend experience and provide structure to advocacy efforts (12, 26). The blended learning approach of this study combined didactic learning and hands-on experience. The survivor advocates offered practical experiences to the master trainers. At the same time, research faculty oversight helped to provide structured mentorship to gain real-world skills in organizing their events, leading outreaches, and engaging stakeholders in their communities. Blended learning with adequate hands-on experiences has been found very effective in advocacies and health promotion, offering opportunities for interactive discussion sessions, applications in real-life settings, and peer support (10, 12). The master trainer-led advocacy programs empower them to take ownership of the cancer awareness efforts. These programs enhance culturally responsive and relevant approaches and efforts that align with the specific community needs.

The outcome of this program showed that over 800 participants from underserved communities were engaged in minimal risk research during the course of the program. Earlier studies have found that collaborating with stakeholders such as advocates and community scientists to lead participant recruitment for studies helps to break down barriers to engagement, particularly in underrepresented and historically marginalized communities (13, 27). These community research advocates understand cultural sensitivities, point out issues regarding mistrust, and use their local networks to encourage participation in cancer awareness programs and research. The engagement and participation in research provide the preliminary evidence of trust building in this study. This program provides the platform to not only improve research participation and diversity but also ensure community ownership of the program, ultimately contributing to more equitable cancer prevention initiatives and research engagement efforts. The need for research advocates in minority communities is both urgent and profound. They are essential in addressing health disparities, building trust, engaging communities, enhancing health literacy, and driving systemic change. By investing in and supporting research advocacy, we can ensure that minority communities have a voice in the research enterprise and that the benefits of scientific discovery are equitably shared. Establishing this framework of advocacy is not only a moral imperative but also a scientific necessity to achieve comprehensive and inclusive health outcomes.

This study has limitations. First, the outcomes presented are not generalizable to other populations since this was a program targeted at IAD. Secondly, the details of the sociodemographic status of the participants of the cancer awareness program were not obtained. Thus, we were unable to provide the demographic characteristics of the participants in cancer awareness programs. Thirdly, most of the Master trainers were from Florida; thus, the outcome of this program may not be generalizable to larger immigrant populations. Fourthly, comments and feedback from the participants of cancer advocacy led by the Master trainers were not comprehensively analyzed because not all participants of the events provided feedback. In addition, the study is only able to provide preliminary evidence of how the IAD could be engaged in cancer programs and research; continuous engagement and programs are necessary to continue trust building between healthcare providers/research centers and community members.

IAD can be trained to effectively lead advocacy programs in immigrant communities, using the train-the-trainer approach. The success of the program lies in grassroots mobilization and inclusiveness, which gives members of the community equitable opportunities to have a voice and sustainable program outcomes. The feasibility of a ToT model of cancer advocacy is not only a moral imperative but also a scientific necessity to achieve comprehensive and inclusive health outcomes. Although the results indicate short-term implementation success, additional research is needed to determine the long-term sustainability of the cancer awareness program, continuous research participation, and cancer outcomes.

Future studies should identify the organizational and community-level factors that facilitate the sustainability of the advocate-led implementation of community outreach and research navigation in IAD communities.

## Data Availability

The original contributions presented in the study are included in the article/Supplementary Material, further inquiries can be directed to the corresponding author.
